# A Comparative Evaluation of the Anxiolytic Effect of Oral Midazolam and a Homeopathic Remedy in Children During Dental Treatment

**DOI:** 10.7759/cureus.31041

**Published:** 2022-11-03

**Authors:** Akshat Agrawal, Nilesh V Rathi, Nilima R Thosar

**Affiliations:** 1 Pediatric and Preventive Dentistry, Private Dental Practitioner and Consultant, Gondia, IND; 2 Pediatric and Preventive Dentistry, Dr. D. Y. Patil Dental College and Hospital, Dr. D. Y. Patil Vidyapeeth, Pune, IND; 3 Pediatric and Preventive Dentistry, Sharad Pawar Dental College and Hospital, Datta Meghe Institute of Medical Sciences (Deemed to be University), Wardha, IND

**Keywords:** oral medicine, dentistry, children, homeopathy, midazolam

## Abstract

Background

Midazolam is recommended by many health standards. However, there is no compelling evidence that midazolam has anti-anxiety effects in children. Homeopathy can be one of the mainstays for effective child management while reducing negative side effects. The aim of the study was to evaluate the anxiolytic efficacy of midazolam (oral) and homeopathic remedies in children during dental treatments.

Methodology

The current ex-vivo study was conducted in the department of Pedodontics and Preventive Dentistry department of a private dental institution. A total of 48 children aged four to 14 years were selected based on the inclusion and exclusion criteria. The participants were evenly and randomly divided into groups A and B using the lottery method. Group A: 20 minutes previous to the treatment, a right blend of an equal volume of 0.5mg/kg injectable solution of midazolam hydrochloride. Group B: Received Aconite napellus (homeopathic remedy).

Results

During anxiety, the hypothalamic pituitary adrenal axis gets activated which causes a release of body fluids including salivary cortisol levels. Salivary amylase also responds quickly during stress and anxiety by increasing its levels.

Midazolam is used in Dentistry to reduce anxiety as it is able to reduce salivary cortisol and amylase levels. Aconite napellus being homeopathic remedy is useful in Dentistry to reduce salivary cortisol and amylase levels which is observed in the present study. There was a decrease in salivary cortisol and amylase concentrations following midazolam (8.51 ± 6.7) (41.48 ± 23.8) and Aconite napellus (homeopathic remedy) (7.53 ± 5.2) (37.08± 22.8) administration, as well as a decrease in heart rate, systolic and diastolic blood pressure. Furthermore, all of the differences were statistically significant (p<0.05).

Conclusion

In children with behavioral difficulties, homeopathic remedy was marginally more successful than oral midazolam in lowering anxiety during dental treatment.

## Introduction

Severe levels of anxiety can hamper a person’s capacity to perform the activities of regular life. It is a continuous sense of fright, nervousness and forthcoming calamity and uneasiness [1]. Dental anxiety (DA) is a negative emotional state experienced by dental patients that is excessive and irrational [2]. Un-cooperation in Pediatric dentistry is characterized by two key aspects: DA and behavioral difficulties in children [3]. It is very common with a high prevalence rate in children aged 4-18 years old and is linked with higher toothache experience, greater occurrence of carious and missing dentition along with the poor oral health-related quality of life [4]. Such patients may be difficult to treat, take longer, and have behavioral issues, resulting in a negative experience for both the patient and the dental practitioner [5]. If patients are not appropriately addressed, it is easy to establish a "vicious cycle" of dental anxiety [6]. Given the negative implications of dental anxiety, it is vital that individuals with this condition receive adequate treatment [5].

While traditional psychological strategies are the cornerstone of behavior control in pediatric dentistry, children with severe dental anxiety are unable to deal with dental treatment using these techniques alone [3]. As a result, pharmacological approaches are used as a supplement to improve child cooperation and promote successful and efficient dental treatment. There remains deficient verification for first-rate preference of sedatives in pediatric patients. However, data has identified midazolam, a short-acting benzodiazepine, as one of the most commonly recommended medicines for dental treatments in children [7].

Its properties like anterograde amnesic effects, anxiolytic and oral administration make it potentially an ultimate sedative agent to be used in Pediatric Dentistry [8]. There are recognized adverse effects, as with any other medicine, ranging from regularly reported minor effects to rarer but more serious negative effects. These could be connected to the dose, the route of administration, or the patient's age. Transient hypoxia, nausea, hiccough, headache vomiting, enuresis, vertigo, hallucinations, hypersalivation, diplopia, dizziness, and behavioral problems are all common adverse effects or paradoxical reactions. Cardiac arrest, heart rate fluctuations, and anaphylaxis are all serious side effects. Therefore, oral health care now a day is concentrated on holistic techniques like acupuncture, Ayurveda, homeopathy and naturopathy in the treatment of diseases and ailments affecting the orofacial structures. As an element of holistic dentistry, homeopathy offers effectual patient management at the same time curtailing undesirable effects [9]. Problems in Teething, toothache, surgical trauma, dental abscess and uneasiness or apprehension are associated with pain or discomfort and can produce anxiety in children and such anxiety doesn’t respond to long-established therapeutic approaches are among the conditions for which homeopathy is regarded effective [10]. Anxiety, as well as other mental health issues like depression and sleeplessness, are among the most prevalent reasons people seek therapy with alternative therapies like homeopathy [1]. In homeopathy, preparations of Aconitum napellus, Coffe cruda, and Gelsium are often used to treat anxiety and other related problems [11]. However, the evidence-based research in dental homeopathy is minuscule and recent systematic reviews and meta-analyses show inconclusive results on the use of homeopathy in dentistry [12,13]. Allopathy remedies give quick response in reducing anxiety but is associated with side effects. Homeopathy remedy can be a safe and economical remedy with minimal or without side effects if it has to be used in children. Depending upon the role of the anti-anxiety effect of Aconitum napellus, its effect in children during dental treatment was considered to be evaluated. Generally, the time allotted for performing dental treatment procedures in a child should be of short time as a child may become restless during the long-term procedure. Therefore, in the dental context for children, the anxiolytic effect of any anti-anxiety drug may have an effect starting from 30 minutes up to two hours for children. Instead of assessing the duration of drugs for the present study, it was decided to conduct a study and assess the anxiolytic effect of midazolam (oral) and the homeopathic remedy in children during dental treatment by evaluating the salivary cortisol, salivary amylase levels and heart rate, blood pressure as well.

## Materials and methods

The current ex-vivo investigation was carried out in a private dental institution's department of Pedodontics and Preventive Dentistry. Prior to the study, Institutional Ethical Committee approval was obtained vide letter number DMIMS(DU)/IEC/2015-16/1521.

The sample size was estimated using the nMaster program (version 2.0, Christian Medical College, Vellore, India). The effect size chosen was 0.4, which was derived from the prior research's mean and standard deviation [14], the error probability was 0.05, the power (1-β probability) was 0.80, and N2/N1 was one. A total of 48 samples were gathered, with 24 in each group.

A total of 48 children aged four to 14 years selected based on the inclusion and exclusion criteria who never had a previous dental appointment and were visiting the Department of Pedodontics and Preventive Dentistry were chosen. All of the participants' parents signed a written informed consent form.

Inclusion criteria for the study were a child with American Society of Anesthesiologists (ASA) I and II who are healthy with mild systemic disease, Frankl II (negative reluctant to accept treatment, uncooperative, some evidence of negative attitude but not pronounced) and Frankl III (positive acceptance of treatment; at times curious, willingness to comply with the dentist, at times with reservation but patient follows the dentist’s directions cooperatively), requiring preventive or restorative dental treatment.

Children with ASA III and IV having severe systemic disease and threat to life, Frankl I with definitely negative behavior and Frankl IV with definitely positive behavior, History of any medication in last three months, requiring dental treatment which could provoke pain were excluded.

Using the lottery approach, the participants were evenly and randomly divided and assigned to groups A and B. Group A: 20 minutes previous to the treatment, a right blend of an equal volume of 0.5mg/kg injectable solution of midazolam hydrochloride (Ampule 1mg/1mL mezolam, Neon Laboratories, Mumbai, India). Group B: Received aconite napellus (homeopathic remedy) at 30c, 60 minutes prior.

Assessment of anxiety: The heart rate, blood pressure, salivary cortisol, and salivary alpha-amylase levels of all the children in both groups were measured at baseline and again during treatment by measuring heart rate, blood pressure with an automatic blood pressure monitor (OMRON HEM-7121) and salivary cortisol and salivary alpha-amylase levels were also measured. In order to collect a saliva sample, patients were asked to seat comfortably for five minutes with their eyes open and their heads tilted slightly. Patients were then asked to spit the saliva which was accumulated on the floor of the mouth into a wide-mouth sterile container and was then transferred to Eppendorf tubes. Samples were placed in a freezing box and sent to the Central research laboratory where they were stored at -20°C in the refrigerator till further processing. The estimation of salivary cortisol (ng/mL) was done by Elisa Salivary Cortisol Kit (The EiAsy™, Diagnostics Biochem Canada Inc., Canada). As part of the specimen pre-treatment technique, the frozen salivary samples were thawed and centrifuged at 2,500 rpm/min for 5 minutes, as recommended by the manufacturer. For the measurement of salivary alpha-amylase (U/L), the stored salivary samples were thawed and centrifuged at 2,500 rpm/min for 5 min as part of the specimen pre-treatment protocol. The saliva supernatant was diluted to a concentration of 1:100 (1%) in distilled water before being processed in an RX imola AY 3805 automated biochemistry analyzer (Randox Laboratories Ltd., United Kingdom).

SPSS 17.0, Graph Pad Prism 6.0 version, and EPI-INFO 6.0 version were used in the analysis and p<0.05 was used as the level of significance. Paired t-test and unpaired t-test were the statistical tests employed to analyze the results.

## Results

Table 1 demonstrates that the difference in mean values of parameters between the two groups when evaluated at the baseline, before the administration of the medications, was minor and statistically insignificant (p>0.05). Similarly, differences in both groups undergoing dental treatment were statistically negligible (p>0.05).

**Table 1:** Comparison of parameters before and after the drug administration in both the groups by unpaired t-test.

Parameters	Group	N	Before	P-value	After	P-value
			Mean ± SD		Mean ± SD	
Salivary Cortisol	Midazolam	24	15.89 ± 7.4	0.708	8.51 ± 6.7	0.440
	Homeopathy	24	16.27 ± 6.7		7.53 ± 5.2	
Salivary α Amylase	Midazolam	24	68.79 ± 27.8	0.098	41.48 ± 23.8	0.904
	Homeopathy	24	62.22 ± 19.9		37.08± 22.8	
Heart rate	Midazolam	24	101.33 ± 7.7	0.597	89.04 ± 11.3	0.801
	Homeopathy	24	97.25 ± 11.6		81.13 ± 12.9	
Systolic Blood Pressure	Midazolam	24	124.88 ± 20.1	0.915	105.17 ± 9.6	0.068
	Homeopathy	24	125.50 ± 18.5		103.42 ± 13.8	
Diastolic Blood Pressure	Midazolam	24	85.79 ± 14.7	0.884	75.54 ± 10.5	0.505
	Homeopathy	24	83.17 ± 12.9		69.92 ± 12.1	

Table 2 reflects the mean values of different physiologic parameters measured at the baseline and during the dental treatment in the midazolam group. According to the results, there was a decrease in salivary cortisol and amylase concentrations following midazolam administration, as well as a decrease in heart rate, systolic and diastolic blood pressure. Furthermore, all of the differences were statistically significant (p<0.05).

**Table 2:** Comparison of values of different parameters before and during the dental treatment after the administration of midazolam using paired t-test.

Midazolam		Mean ± SD	95% Confidence Interval		P-value
			Lower	Upper	
Salivary Cortisol	Before	15.89 ±7.4	5.76	9.00	0.001*
	During	8.51 ±6.7			
Salivary α Amylase	Before	68.79 ±27.8	20.86	33.75	0.001*
	During	41.48 ±23.8			
Heart rate	Before	101.33 ±7.7	7.86	16.72	0.001*
	During	89.04 ±11.3			
Systolic Blood Pressure	Before	124.88 ±20.0	9.57	29.85	0.001*
	During	105.17 ±9.9			
Diastolic Blood Pressure	Before	85.79 ±14.7	2.29	18.21	0.014*
	During	75.54 ±10.5			

Table 3 reflects the mean values of different physiologic parameters measured at the baseline and during the dental treatment in the aconite napellus (homeopathic remedy) group. Similar to the midazolam group in the homeopathy group also decrease in the salivary cortisol and amylase along with a decline in hemodynamic parameters were noted and these were again statistically significant (p<0.05).

**Table 3:** Comparison of values of different parameters before and during the dental treatment after the administration of homeopathy using paired t-test.

Homeopathy		Mean ± SD	95% Confidence Interval		P-value
			Lower	Upper	
Salivary Cortisol	Before	16.27 ± 6.7	6.76	10.71	0.001*
	During	7.53± 5.2			
Salivary α Amylase	Before	62.22 ± 19.9	17.80	32.49	0.001*
	During	37.08 ± 22.8			
Heart rate	Before	97.25 ± 11.7	10.18	22.08	0.001*
	During	81.13 ± 12.9			
Systolic Blood Pressure	Before	125.50 ± 18.5	15.69	28.47	0.001*
	During	103.42 ± 13.8			
Diastolic Blood Pressure	Before	83.17 ±12.9	8.17	18.33	0.001*
	During	69.92 ±12.1			

In both groups, the mean difference of different physiologic parameters obtained at baseline and during dental treatment is shown in Table 4. The comparison of the values indicates that the administration of aconite napellus (homeopathic remedy) produced slightly higher anxiolysis than the administration of midazolam for all parameters except salivary amylase and these differences were found to be statistically insignificant (p>0.05) when compared.

**Table 4:** Comparison of mean differences in parameters before and after giving the drug in both the groups by unpaired t-test.

Parameter	Group	Mean difference	SD	P-value
Salivary Cortisol	Midazolam	7.38	3.84	0.564
	Homeopathy	8.74	4.68	
Salivary α Amylase	Midazolam	27.31	15.27	0.499
	Homeopathy	25.14	17.39	
Heart rate	Midazolam	12.29	10.49	0.258
	Homeopathy	16.13	14.09	
Systolic Blood Pressure	Midazolam	19.71	24.02	0.450
	Homeopathy	22.08	15.13	
Diastolic Blood Pressure	Midazolam	10.25	18.86	0.725
	Homeopathy	13.25	12.03	

## Discussion

Homeopathic medications have gained popularity in various fields due to their low side effects as compared to Allopathic medications. Homeopathic medicine can show side effects if it is consumed in excess. Its side effects are not severe as that of allopathic medicines. But, once the homeopathic drug is stopped, symptoms disappear. Side effects of Homeopathic remedies' can be constipation, dry mouth, decreased sweating, blurred vision, dilated pupils, dizziness, head pain, loss of memory, weakness, confusion, rash, and hives. It is said that if homeopathic medicine is diluted, it can remove the side effects [15]. 

Children experience pain and stress from dental procedures which evokes anxiety in them [16]. Thus, dental anxiety is a major challenge in performing dental treatment in children [17]. Dental phobia or behavior control issues continue to be a deterrent to receiving dental care. The community's impact on this relatively high degree of dental anxiety is far-reaching and multi-faceted.

While behavioral strategies do not include the use of medicines, several children find dental treatment to be difficult to accept. Historically, behavior problems in children have been managed by employing general anesthesia (GA). Although some children will always require this treatment, it is now well-accepted that it should be avoided as much as possible due to the risks involved. Prior to referring a child for Oro-dental treatment, the guidelines for the use of GA in the specialty of Pediatric Dentistry recommend parents consider other potentially safer options [18]. Sedation is considered a promising substitute for GA for behavior management. For behavior management, sedation such as nitrous oxide inhalation sedation may act as an anxiolytic, behavior guidance technique, and as an alternative to GA [19]. Oral midazolam was recognized as one of the few sedatives available whose efficacy in dental treatments for children is supported by data in a recent systematic review due to its high therapeutic index and a large margin of safety [7]. It has anxiolytic, anterograde amnesic, and short-acting properties and can be taken orally or intravenously [3,8]. Despite the availability of a wide range of drugs for the treatment of anxiety disorders, such as benzodiazepines (midazolam), some patients prefer complementary therapies for a variety of reasons, including dependence, addiction, withdrawal anxiety, lack of response, or simply a preference for complementary systems such as homeopathy [14]. Evidence of homeopathy's efficacy in a variety of illnesses has been assessed in several systematic reviews and meta-analyses [13,20]. In each example, the evidence for homeopathy appears to be beneficial when compared to a placebo, but it is mostly equivocal when it comes to specific ailments. Taking a cue from the previously published data, an attempt was made to scrutinize the previously unexplored arena of scientific research through the present study which investigated the anxiolytic effect of homeopathic remedies in children during dental treatment, and it was compared with the anxiolytic efficacy of oral midazolam.

Anxiety is a type of stress, and as such, it has a physiological effect on the body. As suggested by the literature, salivary cortisol levels were utilized to assess stress levels in children in this investigation. Cortisol is a stress hormone that has attracted a lot of interest in psychological and physical health research [21] and salivary cortisol levels accurately reflected circulatory hormone levels, allowing non-invasive evaluation of adrenal activity throughout the day [22]. Anxiety is a fear that arises in response to frightening stimuli, and it is accompanied by and reflected by increases in blood pressure and heart rate [23].

The administration of oral midazolam at a dose of 0.5 mg/kg resulted in significant anxiolysis in children during dental treatment, as evidenced by lower levels of salivary cortisol, salivary alpha-amylase, heart rate, and blood pressure compared to baseline values. Midazolam (benzodiazepine) has an anxiolytic effect by acting on the ascending reticular tract in the midbrain, which maintains alertness, and the limbic system, which supports cognition and mental activities [24]. The reduction of the mean level of salivary cortisol from baseline to after the administration of oral midazolam was observed in the present study. These findings were similar to those reported by Gomes et al. [25], and Pereira-Santos and colleagues [26].

The outcome of the present investigation revealed that the mean level of systolic and diastolic blood pressure was reduced from baseline to after the administration of oral midazolam. The lower levels were within physiologic limits, and they matched the findings of research by Kalibatiene et al. [27] who found that midazolam lowered heart rate and blood pressure from baseline values. However, the effect of midazolam on the vital functions of the patient, including the cardiovascular and respiratory system, was negligible and of no pharmacological significance and was within physiological limits and no adverse effects were observed in any patient at any time during the procedure [27].

Anxiolysis was observed in children receiving aconite napellus (homeopathic medicine) during dental treatment, as evidenced by its capacity to lower salivary cortisol, salivary amylase, heart rate, and blood pressure from baseline. To explain the mechanism of action of homeopathic treatments, several theories have been proposed.

However, no definitive theory has ever been postulated. It is thought to function by boosting the immune system or vital energy, helping the organism to rebalance itself and overcome sick states or disharmonies. Homeopathy's efficiency has been shown in double-blind experiments, but its mechanism of action has never been proven decisively.

The results of the investigation indicated that the reduction in the mean level of hemodynamic parameters occurred after the administration of aconite napellus. Because aconite prolongs sodium influx during the action potential, it exerts a favorable ionotropic impact on the heart [28].

The decrease in mean salivary cortisol levels after aconite napellus administration was consistent with the findings of Merlini et al. [29] who found that fish given a homeopathic complex had significantly lower circulating cortisol levels in an experiment on fish. The decrease in the mean level of salivary alpha-amylase was also detected, which could be ascribed to the activation of the hypothalamic ventromedial nucleus, which controls the ANS and hence suppresses circulation.

The administration of homeopathic remedy showed higher anxiolysis than oral midazolam and the difference in their anxiolytic efficacy was statistically insignificant and was in partial agreement with the observations reported by Lakshmipathy et al. [14] where both diazepam (benzodiazepine) and Puls (homeopathic preparation) showed higher anxiolysis when compared to its control. Even though diazepam has a larger anxiolytic effect than Puls, the authors believe that the homeopathic preparation of Puls is comparable to diazepam. During the course of the present investigation, it was observed that the homeopathic remedy was preferred by both patient and the clinician over oral midazolam as it had a more acceptable taste, and easy administration and it showed higher safety with no known side effects if administered after proper case selection.

Dental procedure-related anxiety in children is dental situation based. Therefore, such anxious children need to be managed with a dental procedure of short duration ranging from 30 minutes to two hours. Aconite napellus, the homeopathic medicine used in the present study was used only for a short duration, i.e., 60 minutes prior to the dental procedure with a minimal dose of 30c. Parameters that are generally studied for assessing anxiety in children include heart rate, blood pressure, and salivary parameters such as cortisol and alpha amylase. Therefore, the present study findings have proved that homeopathic remedies can also be a choice for reducing dental anxiety in children.

## Conclusions

Both groups could decrease salivary cortisol and amylase along with a decline in hemodynamic parameters. Aconite napellus administration produced slightly higher anxiolysis than administration of midazolam for all parameters except salivary amylase. A homeopathic remedy (aconite napellus) was slightly more effective than oral midazolam in reducing anxiety in children during dental treatment and can be used for alleviating dental anxiety as an adjunct to conventional behavior management techniques in children with behavioral problems.
